# Anti-DNA Damage Mechanisms and the Role of Carotenoids, Vitamin A, and Its Derivatives

**DOI:** 10.3390/nu17172721

**Published:** 2025-08-22

**Authors:** Agnieszka Maria Kołodziejczyk, Bolesław Karwowski

**Affiliations:** Nucleic Acids Damage Laboratory of Food Science Department, Faculty of Pharmacy, Medical University of Lodz, ul. Muszyńskiego 1, 90-151 Lodz, Poland; agnieszka.kolodziejczyk@umed.lodz.pl

**Keywords:** vitamin A, retinoic acid, retinol, carotenoids, antioxidant potential, reactive oxygen species, DNA damage

## Abstract

All forms of vitamin A have a similar structure and physiological functions in the body. These compounds can be classified as retinoids, including moieties with a common structure of four isoprenoid units of natural or synthetic origin. Vitamin A is generally uptake from products of animal origin (retinol and its derivatives) or from plants as provitamin A (carotenoids). Vitamin A is fat-soluble, so it is easily absorbed and transported in the body. The main storage sites are the liver and adipose tissue. Excessive amounts of the vitamin may lead to the development of different abnormal processes in the human body. Apart from being crucial for retina conditions and functions and the immune system, vitamin A is also deeply involved in DNA repair mechanisms. Its antioxidant nature helps to reduce the oxidative damage to DNA by neutralizing free radicals and thus decreasing the oxidative stress. On the other hand, vitamin A deficiency leads to lower antioxidant enzyme activity, which results in the weakening of the defense system against free radicals. This study aims to elucidate the mechanisms of DNA repair and determine the role of carotenoids, vitamin A, and its derivatives as contributing factors in this process. This review synthesizes the current knowledge on the dual role of vitamin A in DNA integrity by examining the conditions under which it acts as a genotoxic agent versus a facilitator of DNA repair. This article also discusses the role of vitamin A in inhibiting oxidative stress and its anti- and pro-cancer impact.

## 1. Introduction

**Figure 1:**
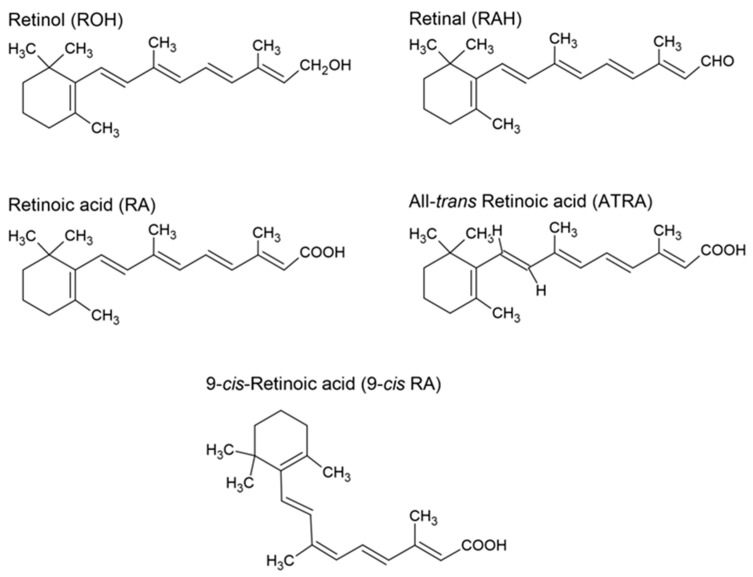
Structures of retinol, retinal, retinoic acid, all-*trans* retinoic acid, and 9-*cis* retinoic acid.

Vitamin A is a non-polar hydrophobic molecule, an isoprene derivative [1]. It is a biologically active, fat-soluble organic molecule [2] that contains a six-membered β-ionone ring with a side chain comprising two isoprenoid units [3]. Non-carotenoid precursors and metabolites of vitamin A include retinol, retinal, retinyl esters, and retinoic acid [1]. The alcoholic (hydroxyl) form of vitamin A is retinol, which needs two transformations to be converted into retinoic acid, whereas the other form of vitamin A derivative, retinal, needs only one. The chemical structures of different forms of vitamin A are presented in Figure 1.

**Figure 2:**
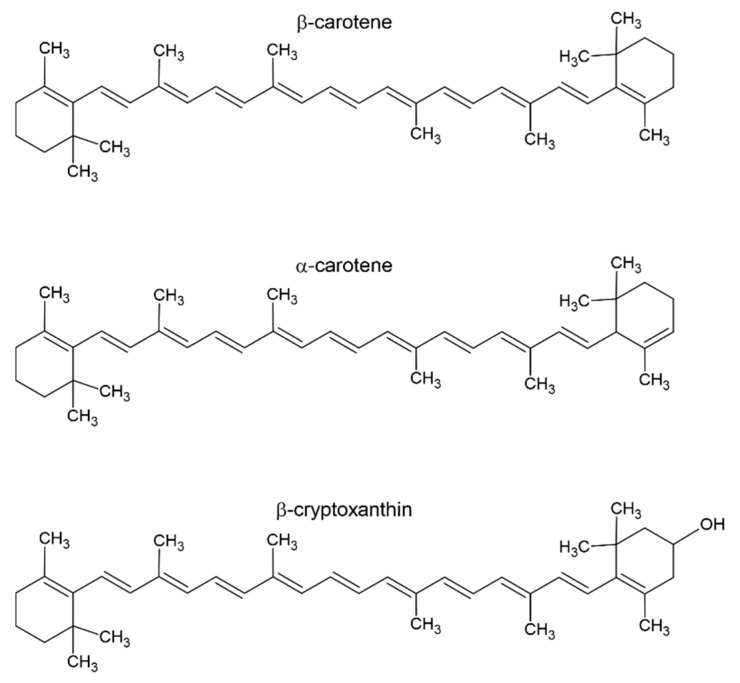
Structures of β-carotene, α-carotene, and β-cryptoxanthin.

Since human body cells are unable to produce vitamin A, it must be obtained from diet, either as provitamin A carotenoids or as preformed vitamin A [2,4,5,6]. Only β-carotene, α-carotene, and β-cryptoxanthin are found in substantial quantities in the human diet out of the more than 50 provitamin A carotenoids [7]. Figure 2 displays the chemical structures of a few chosen carotenoids. Lutein, an alcohol derivative of α-carotene, and lycopene, an organic carotenoid—a natural pigment found in fruits and plants, are also noteworthy. Lutein is currently used as a hormone drug [8] and lycopene is being studied for its role in cancer [9].

Vitamin A is crucial for several physiological functions such as vision [10], gene transcription, and skin cell differentiation [2,11,12]. Also, provitamin A positively affects the immune system by increasing the number of T and B lymphocytes [11]. Consequently, it regulates the immune response and supports the defense mechanisms of the body. Numerous studies [10,13] indicate the contribution of vitamin A as part of the photopigment in visual phototransduction, i.e., the first molecule in the process of converting photons of light into electrical signals. Also, 11-*cis*-retinal, a derivative of retinal, is a component of rhodopsin (light-sensitive pigment), a protein present in the rods of the retina [14,15]. The large light-induced conformational change in the vitamin A-based chromophore makes it an ideal ligand for membrane receptors. This change allows the photoreceptor protein to distinguish the dark state from the activated state (in the light) [16]. Vitamin A deficiency can lead to night blindness [17]. It is also referred to as a growth vitamin [18,19] because it also contributes to the normal development of germ cells and the proliferation of epithelial cells [20]. It has been reported by Hadi et al. that supplementation with high doses of vitamin A improves the linear growth of children with very low serum retinol levels, and that this effect is modified by age and breastfeeding [19]. The antioxidant properties of carotenoids are also widely recognized. Vitamin A neutralizes free radicals and inhibits the activity of matrix metalloproteinase (MMP), the enzymes responsible for extracellular matrix (ECP) remodeling and degradation. Thus, it increases the efficiency of repair mechanisms after UV exposure, protecting the skin against photoaging [21]. Moreover, vitamin A has shown photoprotective properties [22]. It also simulates the production of epidermal proteins, which contributes to the better development of the keratin layer in the stratum corneum (regulation of keratinization) [23]. It helps maintain “healthy” skin, especially in the case of aging, atopic skin, and in external therapies for psoriasis, keratosis, or acne [24,25]. The positive effect of vitamin A on the skin is also associated with the increased synthesis of glycosaminoglycans (GAGs) and the stimulation of angiogenesis in the papillary layer of the dermis [24].

Numerous studies have indicated the many important functions of carotenoids, vitamin A, and its derivatives. The purpose of this work is to summarize the current knowledge on carotenoids, vitamin A, and its derivatives’ impact on DNA repair, which are largely related to oxidative stress in cells. This review focuses on vitamin A (retinol) and five main vitamin A derivatives: retinal, retinoic acid, all-*trans* retinoic acid, 9-*cis* retinoic acid, and 13-*cis* retinoic acid. Due to their increased presence in the human diet, the review also covers the following carotenoids: α-carotene, β-carotene, and β-cryptoxanthin. Cited articles were identified through a systematic and comprehensive search in Scopus, Web of Knowledge, and Science Direct databases through 30 July 2025. All vitamin A’s major issues (e.g., vitamin A, carotenoids, and retinoic acid) and DNA damage/repair mechanisms, DNA damage/repair roles, clinical trials, genotoxicity, oxidative stress, and tumorigenesis were selected as keywords to find related studies. From the systematic analysis, 102 articles were directly included in the current study. Additional reports were added within the comprehensive studies in the review.

## 2. Absorption and Transport of Vitamin A

**Figure 3:**
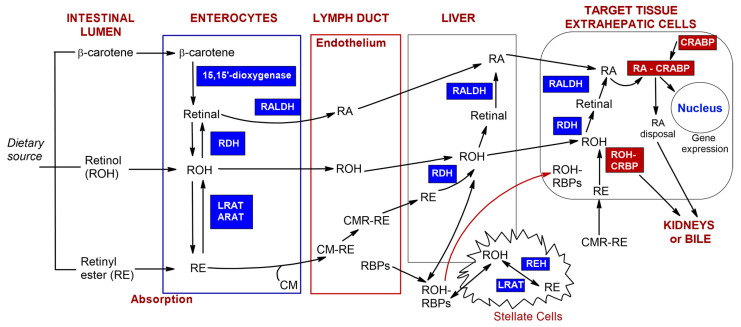
The scheme of absorption and transport of vitamin A and carotenoids prepared based on the literature sources [5,27,28]. Retinol (ROH), carotenoids (e.g., β-carotene), and retinyl esters (RE) from the diet can be metabolized in the intestinal lumen. Depending on their original form, after penetration of enterocytes, they undergo esterification or oxidation. Next, chylomicrons–retinyl ester conjugates (CM-RE) are secreted into the lymphatic system. Subsequently, they reach the blood vessel and subsequently liver, which is the main storage organ for retinoids in the body or target cells. (Other abbreviations: retinaldehyde dehydrogenase (RALDH), retinol dehydrogenases (RDH), lecithin–retinol acyltransferase (LRAT)).

Vitamin A and carotenoids, incorporated into micelles alongside other dietary lipids, penetrate enterocytes [5,26]. The absorption and transport scheme of vitamin A and carotenoids, as presented in Figure 3, is based on sources from the literature [5,27,28].

In the cytoplasm of the intestinal epithelium, β-carotene is oxidized by 15,15′-dioxygenase to form retinal [29]. This is followed by the oxidation of retinal to retinoic acid via retinaldehyde dehydrogenase (RALDH) [27]. Subsequently, retinal can be converted into retinol through the action of retinol dehydrogenases (RDH). The esterification of retinol in enterocytes occurs with the help of enzymes such as acyl-CoA—retinol acyltransferase (ARAT) and lecithin—retinol acyltransferase (LRAT) [5,27,28]. Retinyl esters (RE) are hydrolyzed to retinol via intestinal enzymes or adsorbed as a component of plasma chylomicrons (CMs). CMs are primarily intestinal lipoproteins consisting of triacylglycerol and phospholipid particles, as well as carotenoids, retinyl esters, and other fat-soluble vitamins. These particles enter the intestinal lymph through endocytosis. After combining with chylomicrons (CM-RE), retinyl esters are secreted into the lymphatic system [30]. The subsequent hydrolysis of triacylglycerols and exchange of apolipoproteins result in the formation of chylomicron remnants (CMRs). Almost all of the retinyl esters present in the chylomicrons remain during the conversion process to form CMR-RE complexes. CMR-REs are then delivered to the liver, where they are stored [31]. Retinyl esters, such as retinyl palmitate, oleate, myristate, stearate, and linoleate, are stored in specialized storage cells known as stellate cells [32]. When required by the body, the esters are released from the liver. The enzyme esterase converts retinyl esters into retinol. The RBP receptor (RBPR) enables the transport of retinyl esters or retinol (ROH) to target tissues. Once they reach the inside of target cells, the oxidation stage begins with the involvement of either retinol dehydrogenase (RDH) or alcohol dehydrogenase (ALDH). This is followed by the oxidation of retinal into retinoic acid via enzymes from either the cytochrome P450 (CYP26) or the retinaldehyde dehydrogenase (RALDH) family [33,34]. The activity of retinoic acid and retinol is regulated by cytosolic proteins that bind to either retinoic acid (CRABPI and CRABPII) or retinol (CRBPI and CRBPII) [28]. Intracellular CRBP proteins create a thermochemically favorable gradient for the cellular uptake of retinol when its binding sites are not restricted by ligands [35,36]. CRABP proteins capture free retinoic acid, which is then broken down by enzymes or converted into inactive forms. The resulting products are easily removed by the kidneys and/or excreted in bile [37]. Also, vitamin A can be found embedded in the lipid bilayer of cellular and subcellular membranes [38], where the cyclic carbon ring is likely to be oriented near the polar group region of the lipid bilayer, and its polyene chain extends into the central non-polar region of the bilayer [39]. By contrast, the portion of retinol that is not converted into retinyl esters via intestinal cells is secreted directly into the bloodstream, where it can bind to retinol-binding protein (RBP) [40]. Stored retinoids can be secreted directly from the liver into the blood, in combination with RBP, or they can bind to other transport proteins, such as albumin, in the blood at a later stage. The pharmacokinetics of vitamin A can also be affected by the pharmaceutical intake. Jung et al. [41] have indicated that estrogen and oral contraceptives increase plasma concentrations of retinol-binding protein (RBP), thereby raising blood retinoid levels. Conversely, ethanol consumption is a key factor in the inhibition of vitamin A metabolism. Retinol concentrations are significantly reduced in alcoholic liver disease [42].

## 3. Antioxidant Potential of Vitamin A

Reactive oxygen/nitrogen species (RO/NS) determine a number of molecular oxygen derivatives associated with normal physiological functions, including cell signaling, gene expression, proliferation, immune response, and redox homeostasis [43,44,45]. Reactive oxygen species are produced during oxygen metabolism inside the mitochondrial matrix, and this is their main source [46,47]. Under the influence of various factors, reactive oxygen species levels can rise, reaching non-physiological amounts that endanger the proper functioning of cells. Elevated levels of different reactive oxygen species lead to the formation of DNA damage, denoted as ‘oxidative stress’, which can affect the integrity of nuclear and mitochondrial DNA (*mt*DNA).

A lack of balance between reactive oxygen species production and antioxidant defense systems contributes to oxidative stress [48,49,50]. Reactive oxygen species include the following: $O_{2}^{•-}$ (superoxide anion radical) and conjugated ${HO}_{2}^{•}$ (hydroperoxide radical), ^•^OH (hydroxyl radical), ^1^$O_{2}$ (singlet oxygen), $O_{3}$ (ozone), and $H_{2}O_{2}$ (hydrogen peroxide) [51]. The ^•^OH radical is responsible for the most oxidative damage to DNA.

The specific forms of vitamin A disclose different antioxidant mechanisms. Vitamin A, which belongs to a broad group of vitamins, binds to superoxide radicals, acting as an antioxidant that breaks the chain of reactions even before radicals interact with lipids. The antioxidant activity of vitamin A and carotenoids is conferred by their hydrophobic chain of polyene units, which can quench singlet oxygen, neutralize oxygen radicals, and combine and thus stabilize peroxyl radicals [35,52]. The capacity to stabilize the formation of peroxyl radicals has been observed to increase with the length of the polyene chain [35]. The aforementioned findings point to the action of vitamin A in preventing the formation of hydroperoxides and protecting cells from damage.

By inhibiting peroxidation in both model phosphatidylcholine liposomes and a homogenous solution of methyl linoleate, retinol efficiently removed peroxyl radicals [53]. In addition to neutralizing peroxyl radicals, vitamin A has been proven to undergo direct oxidation by radicals, leading to the formation of 5,6-retinoid epoxide and stabilizing the lipid radical. Also, retinol appears to be effective in eliminating potentially harmful glutathione radicals [53]. It has also been reported that retinol may operate as both an antioxidant and a pro-oxidant, producing reactive hydroxyl radicals [54].

For both hyperoxia-induced kidney failure and hyperglycemia, antioxidant capabilities were exhibited. They included reactive oxygen species removal, antioxidant enzyme activity restoration, improved glutathione levels, decreased malondialdehyde concentrations [55], and the inhibition of advanced end glycation product generation [56]. In turn, Wu et al. discussed the role of oxidative stress in the formation of neural tube defects during early embryogenesis through exposure to high doses of all-*trans* retinoic acid (ATRA) [57]. Additionally [57], increased doses of all-*trans* retinoic acid induced pathophysiological mechanisms in rat embryos, revealed as reduced superoxide dismutase (SOD) and glutathione peroxidase (GSH-px) activity, along with increased levels of malondialdehyde (MDA) and changes at the protein level—increased levels of Bax and caspase-3 and decreased levels of Bcl-2, suggesting a shift toward proapoptotic pathways. Thus, it can be concluded that vitamin A is an important factor in reducing oxidative stress, i.e., one of the primary causes of DNA damage. On the one hand, it acts as an indirect antioxidant since it indirectly affects the body’s antioxidant systems by reducing the level of reactive oxygen species. On the other hand, it also functions as an apoptosis modulator because in severe DNA damage, vitamin A can regulate apoptotic processes, eliminating damaged cells and preventing uncontrolled proliferation.

**Figure 4:**
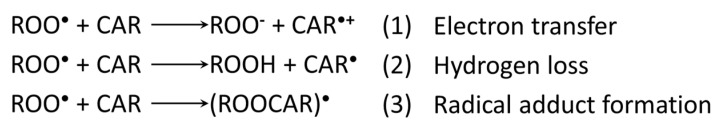
Chemical reactions of free radicals and carotenoids [52].

Additionally, carotenoids, which are precursors of vitamin A, have the ability to eliminate volatile and highly reactive singlet oxygen and superoxide radicals [58,59]. Carotenoids are non-enzymatic antioxidants that convert reactive oxygen species into inactive forms while undergoing agitation themselves. They may engage in three types of interactions with free radicals: radical adduct formation, energy transfer, and hydrogen loss [60]. Figure 4 is a schematic representation of the chemical reaction of carotenoids with free radicals. The first reaction (1) involves electron transfer between a free radical (ROO^•^) and a carotenoid, which results in the formation of a carotenoid radical cation. It could also be hydrogen loss (2), where hydrogen atom transfer leads to the formation of a neutral carotenoid radical. Additionally, radical adduct formation (3) is discussed [61].

The kind of free radical itself has a significant impact on the mechanism and rate constant of carotenoids’ scavenging of these radicals in solution. Carotenoids are broken down/oxidized primarily via the activity of degradation enzymes and antioxidants. During mild oxidative stress, high-molecular-weight products, such as apo-carotenals, are generated as a result of the interaction with reactive species. Depending on the oxidant’s characteristics and concentration, these compounds are further oxidized to short-chain breakdown products [62]. This condition of low/high oxygen concentrations is directly connected to carotenoids’ contentious anti-/pro-oxidant dual actions, which will be investigated further in this review. Other factors that have a significant impact on antioxidant activity include the following: structure, location, and orientation in the lipid bilayer (i.e., incorporation into membranes), ability to interact with other carotenoids or antioxidants, and oxygen concentration and its partial pressure. The term carotenoid structure refers to the size and shape, as well as the physical form, including monomorphic, agglomerated, and isomeric *cis* or *trans* forms. In biological systems, carotenoids rarely exist as monomeric molecules ‘in solution’. They are closely associated with protein or lipoprotein structures. Carotenoids owe their activity to their highly conjugated isoprenyl chains, which allow for the increased resonance stabilization of radicals [63,64]. The concentration of the carotenoid itself can affect the antioxidant capacity, i.e., in high concentrations, the antioxidant activity of carotenoids decreases or can induce pro-oxidant activity. Salerno et al. [65] evaluated the impact of carotenoids’ cleavage products on neutrophil activity. Retinal (0.01–10 μM) induces $O_{2}^{•-}$ synthesis and release in guinea pig macrophages at inadequate quantities while blocking it at higher concentrations (10–100 μM). In pure human neutrophil suspensions, retinal and β-ionone at micromolar concentrations (1 μM) promoted $O_{2}^{•-}$ generation, but not carotenoids lacking a carbonyl moiety. Carotenoids suppressed $O_{2}^{•-}$ generation at doses over 20 μM in the presence of phorbol myristate acetate and chemotactic tripeptide *N*-formyl-Met-Leu-Phe [66]. These various results reflect the carotenoids’ concentration-dependent varied behavior. Oxygen pressure affects the anti/pro-oxidant action of β-carotene. β-carotene serves as a chain-breaking antioxidant in low oxygen conditions, which are common in human tissues. As oxygen tension rises, β-carotene becomes more easily autoxidized and may exhibit pro-oxidant properties [67]. Gupta et al. [68] found that the antioxidant and pro-oxidant effects of α- and β-carotene (0.001–0.1%, *w*/*v*) varied depending on the concentration and in vitro system. Carotenoids exert antioxidant activity at low-oxygen partial pressure (below <150 Torr); however, they may lose these properties or even become pro-oxidants at high oxygen concentrations due to autoxidation processes [52,60]. Carotenoids can exert indirect antioxidant effects by regulating antioxidant enzymes, such as superoxide dismutase (SOD) and catalase (CAT), thus enhancing the body’s defenses against oxidative stress. In summary, at least three parameters are important in determining the rate and type of mechanism for the reactions of carotenoids with different radicals. These are the structure of the carotenoid, the average polarity, and the reactivity of the radical.

## 4. DNA Mutation—From Genomic to Non-Genomic Mechanisms Induced by Vitamin A

DNA mutations can be initiated by endogenous or exogenous factors [69,70]. Endogenous factors include genetic predisposition, while exogenous factors may be divided into different groups such as chemical (e.g., alkylating compounds), physical (e.g., ionizing radiation), pharmacological (e.g., certain drugs), and environmental (e.g., environmental pollutants, smoking, or an improper diet). Among the major endogenous factors causing DNA mutations is the formation of reactive oxygen species at a non-physiological level (approx. 2–3 nmol superoxides/min/mg protein) [71,72,73,74]. Over 100 types of oxidative DNA damage have been identified [75], including the oxidation of nucleic bases, sugar residues, single/double strand breaks (SSBs/DSBs), the formation of baseless/apurin/apyrimidine (AP) sites, deletions and/or translocations of chromosome fragments, and others. To a large extent, oxidative damage is caused by hydroxyl radicals after water radiolysis. The products of water radiolysis, in close proximity to nucleic bases or sugar residues of nucleotides, can react with them, leading to the generation of damage. Factors contributing to DNA damage include the presence of hydrogen peroxide, superoxide anions, and lipid peroxides, which are inert to DNA under natural conditions. However, when converted to hydroxyl radicals in the Fenton or Haber–Weiss reactions [76], these molecules can cause damage to nuclear and mitochondrial DNA (mtDNA), e.g., SSBs and DSBs.

**Figure 5:**
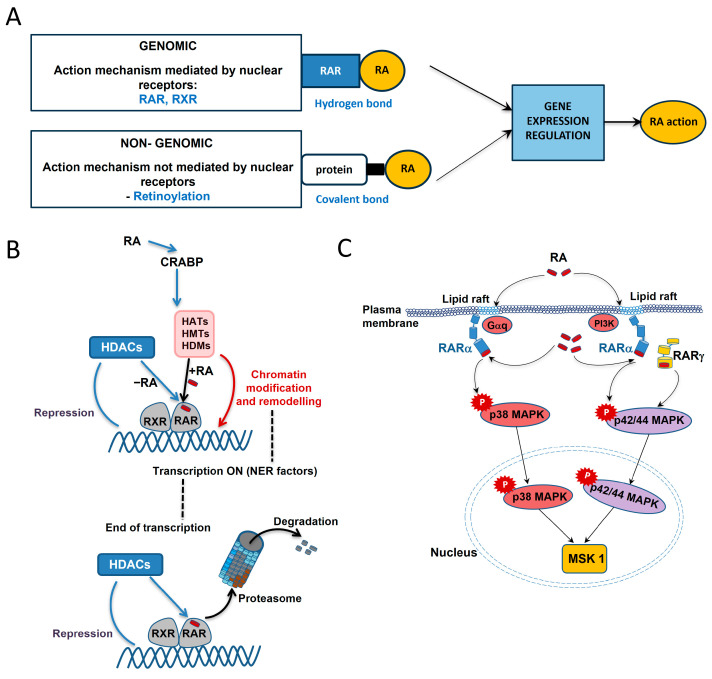
Retinoic acid (RA) action mechanisms [78] (**A**) including genomic–coregulator exchange at RXR/RAR heterodimers [77] (**B**) and non-genomic activation of kinase cascades via an extra nuclear pool of RARs [77] (**C**). For the genomic mechanism, the RAR and RXR nuclear receptors are involved, while retinoic acid and Retinoic Acid Receptors are bound by hydrogen bonds, whereas the non-genomic mechanism is based on retinoylation—a reaction in which retinoic acid and amino acids on intracellular proteins are covalently bound. The gene expression regulation is the result of both mechanisms.

In the case of vitamin A and retinoids, we can distinguish two pathways of action for DNA damage/repair: genomic and non-genomic (extra-nuclear) [77]. These two mechanisms are schematically illustrated in Figure 5A based on the retinoic acid’s action [78].

Various vitamin A forms can bind to nuclear receptors, inducing or repressing the expression of target genes, thus performing genomic action (Figure 5A upper panel and Figure 5B). Retinoic acid is a key regulator of gene transcription through its interaction (hydrogen bonds) with specific nuclear Retinoic Acid Receptor (RARs) and Retinoid X Receptor (RXRs), each of which has three isoforms, i.e., α, β, and γ [79]. First, retinoic acid mobilizes to the nucleus by binding to small cytosolic proteins (CRABP) and then the resulting complex directs retinoic acid to RARs (Figure 5B from the top). They transmit retinoic acid signals in heterodimers with RXRs and function as ligand-dependent transcription regulators. Tanoury et al. found that the DNA-bound RAR subtype is linked to corepressors that operate as adapters, attracting high-molecular-weight complexes with histone deacetylase (HDAC) activity when there is no ligand present [77]. Large complexes with a variety of enzymatic activities are recruited in an ordered and coordinated manner by coactivators. These complexes include histone acetyltransferases (HATs), histone methyltransferases (HMTs), histone demethylases (HDMs), and DNA-dependent ATPases. All of these complexes modify and modulate the chromatin structure. This includes the formation of markers or binding sites that form a “histone code” read by specific effectors. Upon retinoic acid binding, the RAR-RXR heterodimer recruits coactivators and activates the transcription of target genes, also affecting the efficiency of other signaling pathways (“crosstalk”) [79,80].

According to a study by Talib et al. [81], the transcription mechanism binds sequentially to the promoter’s nucleotide excision repair (NER) components in order to provide the best possible histone modifications. The termination of the transcriptional response is associated with coregulators such as receptor-interacting protein 140 (RIP140), recruited by liganded RARs. RIP140s inhibit RAR transcriptional activity by recruiting histone deacetylase (HDACs). RARs are degraded by the ubiquitin–proteasome system. The absence of RAR-RXR heterodimer ligands or presence of certain antagonists may result in the suppression of specific target genes. This is due to the (presumably gene-specific) recruitment of complexes containing histone deacetylase bound by corepressors (CoRs) [82]. The consequence is chromatin compaction, the deacetylation of histones, and the inhibition of target gene promoter regions.

It is estimated that all-*trans* retinoic acid affects the activity of up to 500 genes and has anti-inflammatory, anti-tumor, and cell proliferation inhibitory effects [83,84]. The mechanism of all-*trans* retinoic acid action is mediated by binding to RAR α, β, and γ, but this leads to efficient gene expression only via RARβ, RARγ, and RXR [85]. The cited observation [85] was made in the context of topical tretinoin, a dermatological substance that has many desirable properties. It modulates epidermal cell proliferation and differentiation, stimulates new collagen formation, reduces inflammation, and stimulates fibroblasts, among others. Burzynski et al. [84] discussed whether vitamin A can affect the molecular pathways involved in maintaining redox homeostasis in acute pancreatitis (AP) and chronic pancreatitis (CP). It is concluded that there is insufficient data to determine whether vitamin A administration can relieve acute pancreatitis and/or chronic pancreatitis symptoms. However, the compound has been shown to exert antioxidant and protective effects through the activation of specific RARs, such as RARα, RARβ, and RXR. The post-translational effects of specific nuclear receptors are also important. Xu et al. have shown that post-translational modifications of RARα, such as phosphorylation, sumoylation, and ubiquitination, play a significant role in the action of retinoic acid on DNA binding, transactivation, and the degradation of RARα [86].

From the viewpoint of gene mutations, microRNAs (miRNAs) are also an important area of study. These are small non-coding RNAs that act as endogenous regulators of gene expression at the post-transcriptional level [87]. Through numerous interactions with target mRNAs, microRNAs exert pleiotropic biological effects, participating in many physiological pathways of cells both during development and in adulthood [87]. Their role has been widely evidenced in the context of metabolic disorders and cellular programming mechanisms. A particular area of research remains microRNAs targeting nuclear receptors—Retinoic Acid Receptors (RARs) and Retinoid X Receptors (RXRs). Previous analyses have indicated that their function in regulating these receptors is poorly understood and is a prospective direction for further research [88]. The focus of future studies should be on understanding whether and how the action of retinoids and microRNAs can trigger a cascade of dynamic events that result in the precise, specific control of gene expression. The research literature suggests that microRNAs play an important role in the precise fine-tuning of retinoid-regulated developmental and cell differentiation pathways [88]. Nevertheless, only a few microRNAs directly interact with the main Retinoic Acid Receptors, Retinoid X Receptors, and proteins involved in retinoic acid signaling. Bioinformatic studies [89] have shown that microRNAs can target the main Retinoic Acid Receptors and Retinoid X Receptors through conserved sequences present in mammalian and vertebrate microRNA families, indicating a potential mechanism of action. Kim et al. [90] investigated the effect of β-carotene on microRNAs, histone acetylation, and global DNA methylation in colorectal cancer stem cells (CSCs). MicroRNA sequencing analysis showed that β-carotene modulates the expression of microRNAs associated with histone acetylation. An increase in the level of histone H3 and H4 acetylation was observed after treatment with β-carotene. In addition, DNMT3A mRNA expression and global DNA methylation were reduced, indicating that β-carotene may influence epigenetic modifications, exhibiting anti-cancer activity against colorectal cancer stem cells [90]. In another article [91], the effect of the Western diet of the mother and β-carotene supplementation in early life on the epigenetic remodeling of liver microRNA in mouse offspring was evaluated. The experiment was conducted in two groups: control (placebo) and with β-carotene supplementation during breastfeeding. The results indicate gender-dependent changes in the expression profile of liver microRNA [91], which may be relevant for further research on the long-term effects of β-carotene supplementation in the postpartum period on the metabolic development of offspring in the context of an unbalanced maternal diet. MicroRNAs are a key element in gene expression regulation, and their interactions with Retinoic Acid Receptors and the epigenetic modifications induced by β-carotene open up new perspectives in research on the mechanisms of development, differentiation, and cancer therapy.

Non-genomic action (Figure 5A bottom panel and Figure 5C), on the other hand, relies on the phosphorylation of certain targets to regulate the signaling pathway. This action causes protein kinases and phosphatases to respond quickly, regulating cellular activities and causing a physiological response to a stimulus [92,93,94,95]. The scheme of non-genomic action in the example of retinoic acid is presented in Figure 5C [88]. The extra-nuclear action occurs through interactions with RARs on the cell membrane. Depending on the cell type, RARαs in the lipid rafts have been found to interact with Gαq proteins and activate kinase pathways, or may directly interact with another PI3K kinase. This leads to retinoic acid signaling pathways that cause the activation of mitogen-activated protein kinase (e.g., p38 MAPK or p42/44 MAPK) cascades [96,97,98]. RARα and MAPK then enter the cell nucleus and phosphorylate mitogen- and stress-activated kinase 1 (MSK1). Next, a number of nuclear components implicated in the expression of retinoic acid target genes are phosphorylated by MSK1. In a study [97], it was demonstrated that introducing RA into SH-SY5Y neuroblastoma cells activates the PI3K and ERK1/2 MAPK signaling pathways quickly and non-genomically without the need for newly produced proteins or the transcription of new genes.

## 5. An Overview of DNA Damage/Repair’s Role in Carotenoids and Vitamin A

The appearance of oxidative damage and its accumulation can initiate DNA mutations resulting in many diseases [99]. Damage occurring in the area responsible for encoding the genes crucial for proper cell function (e.g., tumor suppressor genes) can lead to mutations that impair cell function or induce cancerous transformation. Thus, the reduction in ROS is very important and can be directed as a preventive measure in many diseases such as cardiovascular diseases [100,101] and cancer [102]. In human studies, the results often present correlations between vitamin A, its derivatives, or carotenoids (in supplemented doses) and DNA damage markers. Astley et al. have shown that supplementation with carotenoids and carotenoid-rich foods modulates DNA damage [103]. This has been found to achieve DNA repair equilibrium in human lymphocytes. The study examined the decay rate of H_2_O_2_-induced single-strand breaks (SSBs) in peripheral blood lymphocytes (PBLs) [103]. A comparative analysis was performed for supplementation with carotene capsules, which led to improved cellular regeneration after oxidative challenge. On the other hand, carotenoid-rich foods did not produce the same effect [103]. Park et al. [104] showed a negative correlation between the levels of α- and β-carotene and single-strand breaks induced ex vivo in lymphocytes via H_2_O_2_. This study included patients with coronary heart disease and a control group. In the next article [105], the urinary 8-hydroxy-2′-deoxyguanosine (8-OHdG) concentration was negatively correlated with the retinol concentration in lung cancer patients, though this correlation was not observed in the control group. On the other hand, vitamin A was positively associated with 8-hydroxy-2′-deoxyguanosine (8-OHdG) in patients with esophageal cancer, but not in controls [106]. Diaz-Garcia et al. [107] showed that for a mother–newborn population, there is a correlation between reduced levels of 8-OHdG and increased vitamin A supplementation (*p* < 0.01). In contrast, studies of men and women aged 40–64, including smokers, found no correlation between oxidative DNA damage and the consumption of the antioxidant vitamins A, C, E, and β-carotene [108]. Another study summarized upper gastrointestinal cancer biomarkers one year after supplementation with a cocktail containing vitamin A, riboflavin, zinc, and selenium [109]. A reduction in the frequency of micronuclei (*p* < 0.001) and DNA adducts of carcinogens, which are considered endpoints for intervention in chemoprevention trials, was obtained [109]. Collins et al. [110] conducted a study on a male and female population aged 25–45 to examine the effect of vitamin E (first 4 weeks, 100 mg/day) followed by carotenoid supplementation (12 weeks, 15 mg/day) in the selected configurations: a mixture of α- and β-carotene (in proportions of 30 and 60%, respectively), lutein, and lycopene (along with 10% β-carotene). The results from the article [110] proved an inverse correlation between the frequency of oxidized bases in lymphocyte DNA and the concentration of carotenoids in the blood. Similarly, for xanthophyll carotenoids, including lutein and β-cryptoxanthin, an inverse correlation was observed between their plasma levels and urine 8-OHdG levels [111]. Zhao et al. [112] conducted a study on the effect of various carotenoids, including β-carotene, lutein, and lycopene, each in a daily dose of 12 mg or in a mixture of 4 mg each, on oxidative DNA damage in a group of postmenopausal women (aged 50–70). The results indicate that, as early as day 15 of supplementation, endogenous DNA damage is significantly reduced (*p* < 0.01) in the group supplemented with a mixture of carotenoids and β-carotene alone. Meanwhile, the placebo group showed no significant changes. Pool-Zobel et al. [113] investigated the effect of consuming foods rich in carotenoids on the protection of DNA damage and oxidative DNA damage in a population of men aged 27–40. The group under study was given food at specified intervals. First, there were two weeks without supplementation, then foods containing 40 mg of lycopene (weeks 3 and 4), followed by 22.3 mg of β-carotene and 15.7 mg of α-carotene (next two weeks), and in the final stage, 11.3 mg of lutein (last two weeks) were given. A reduction in the endogenous levels of DNA strand breaks in lymphocytes was observed after the consumption of foods rich in lycopene, lutein, α-carotene, and β-carotene, while oxidative DNA damage was reduced via α- and β-carotene [113].

Although many research reports indicate the contribution of carotenoids [103,104,110,111,112,113] or vitamin A [105,107,109] in reducing DNA damage, these studies were conducted in various supplementation configurations (high heterogeneity in study design), often on small populations ranging from 23 to 43 individuals [103,104,105,106,110,111] and in a wide age group. The long-term effects of supplementation have not been studied, i.e., it is not known how long the effect of reducing DNA damage markers persists in the body. This review also mentions studies [106,108] in which no reduction in DNA damage after vitamin A supplementation was demonstrated. Hence, it is not possible to clearly determine at what doses of carotenoids, vitamin A, or their derivatives, or in what disease states DNA repair processes will be initiated. Nevertheless, the present review indicates that carotenoids, vitamin A, and its derivatives are potential health-promoting factors for which, with additional clinical studies, knowledge about the initiation of DNA repair processes after their supplementation could be confirmed.

Studies on the effects of vitamin A and its derivatives in animal models demonstrate their protective effects on DNA repair mechanisms. For instance, retinoic acid decreased the number of single-strand breaks (SSBs) in the liver DNA of rats treated with *p*-dimethylaminoazobenzene (DAB) [114]. Morin et al. demonstrated that administering retinyl acetate for 24 h reduced the level of 8-oxo-7,8-dihydro-20-deoxyguanosine (8-oxodG) in the DNA of rat leukocytes [115]. Liu et al. assessed the effect of β-cryptoxanthin supplementation on cigarette smoke-induced DNA oxidative damage (8-hydroxy-2′-deoxyguanosine (8-OHdG) levels) in male ferrets [116]. In ferrets, β-cryptoxanthin supplementation increased the plasma and lung levels of β-cryptoxanthin in a dose-dependent manner, whereas cigarette smoke exposure decreased these levels. Additionally, β-cryptoxanthin significantly reduced lung squamous metaplasia and smoke-induced inflammation, as evidenced by lower 8-OHdG levels [116]. As demonstrated by Wolterbeek et al. [117], vitamin A and β-carotene cause unscheduled DNA synthesis in the hamster tracheal epithelium in organ cultures exposed to benzo[a]pyrene (B[a]P). This effect depends on the dose of B[a]P and concentration of vitamin A. It has been reported that both vitamin A and β-carotene increase DNA repair activity, which lowers the amount of B[a]P-DNA adducts. This may lead to processes underlying vitamin A-mediated cancer prevention. Aissa et al. [118] investigated the effect of pure and microencapsulated β-carotene (mBC) on Wistar rats at doses of 2.5 and 5 mg/kg for a period of 14 days in genotoxicity tests with doxorubicin (a dose of 16 mg/kg causes chromosome damage in rodents). The results of the micronucleus test showed that mBC is anti-genotoxic at the higher dose, while the “comet assay” showed that mBC had a protective effect in the liver. The authors suggest that the lack of effects for pure β-carotene is probably related to the reduced bioavailability of the carotenoid, which was modified by the microencapsulation process [118].

Bhagavathy et al. [119] investigated the effects of lutein and β- and α-carotene present in extracts of the green algae *Chlorococcum humicola (C. humicola)* on human lymphocytes through chromosomal aberration (CA), sister chromatid exchange (SCE), and micronucleus assay (MN). The administration of benzo(a)pyrene (B(a)P) resulted in an increase in CA and SCE, but incubation with carotenoids resulted in their reduction. Also, a decrease in MN (*p* < 0.05) was observed after the incubation of lymphocytes with carotenoids. Murata et al. [120] revealed that vitamin A and retinal induced cellular DNA cleavage. The studies were performed for HL-60 (suspension cell line from a patient with acute promyelocytic leukemia) and its H_2_O_2_-resistant HP100 clone. Both vitamin A and its derivative induced the formation of 8-oxo-7,8-dihydro-2′-deoxyguanosine (8-oxodG) in HL-60 cells, though not in HP100 cells, which indicates the involvement of H_2_O_2_ in cellular DNA damage. Also, studies have been performed using ^32^P-labeled DNA isolated from the human p53 tumor-suppressor gene and the c-Ha-ras-1 protooncogene. The results they obtained revealed that retinol and retinal induced Cu(II)-mediated DNA damage, which was prevented by catalase [120]. In turn, the article [121] revealed that β-cryptoxanthin protects against H_2_O_2_-induced damage in in vitro studies (HeLa and Caco-2 human cells). The incubation of H_2_O_2_-treated cells with β-cryptoxanthin led to a doubling of the rate of strand break rejoining and had a similar effect on the rate of oxidized purine removal via base excision repair.

## 6. Tumorigenesis—The Role of Carotenoids, Vitamin A, and Its Derivatives

The effects of carotenoids, vitamin A, and its derivatives are related to their DNA repair mechanisms, which reduce oxidative stress and also induce apoptosis or ferroptosis [81,122,123]. Oxidative stress is a major element in the development of cancer. It is the result of an imbalance between the activity of prooxidant molecules and the antioxidant defense system [50]. Oxidative stress can cause cancer through a variety of processes, including the disruption of essential metabolic pathways and oxidation of nuclear DNA, which results in genetic alterations and instability. Carotenoids, vitamin A, and its derivatives have anti-DNA damage characteristics mostly due to their antioxidant activity, which helps to neutralize reactive oxygen species and reduces oxidative stress. Thus, they may contribute to the reduction in tumorigenesis [124,125]. Reactive oxygen species can disrupt normal physiological processes, such as signal transduction, protein synthesis, and cell division. This may further increase the risk of cancer. Factors directly influencing tumorigenesis include hereditary and acquired genetic changes, chromosomal rearrangements, and various epigenetic factors.

Didier et al. [50] studied the relationship between reactive oxygen species and antioxidants in cancer genesis. Tumor formation may be separated into three stages: initiation, promotion, and progression. DNA may be destroyed by a range of physical factors, including UV radiation and chemical compounds. The cell then maintains the mutation in its genomic DNA and becomes an initiated cell. Spontaneous mutations produced by inadequate DNA repair can also promote the creation of initiated cells. Oxidative stress is the fundamental driving force for initiated cell growth [72]. The modified cell then enters the promotion phase, where it preferentially expands and forms a preneoplastic lesion. The last phase involves lesion replication, which causes the tumor population’s cells to acquire mutations. This can provide the mutation with a selective advantage—increased growth factor production—and cause the cell to become dominant in the population (clonal selection process) [50]. Retinoic acid exhibits some anti-tumor activity through the modulation of gene expression, which can induce the differentiation and/or apoptosis of cancer cells and the inhibition of tumor promotion in chemically induced tumors. The ability to regulate growth as well as induce differentiation affects the cell culture of many cancer cell lines, where the main action can be distinguished for a specific RAR or RXR. It is noteworthy that the recent literature reports [126] have indicated the occurrence of retinoic acid anti-tumor effects that are not mediated by RARs or RXRs. According to Takahashi [126], retinol, retinoic acid, and retinyl palmitate have an impact on the development of resistant human cancer cells, including pancreatic cancer cells (MIA Paca2, JHP-1), gallbladder cancer cells (NOZ C-1), and cholangiocarcinoma (HuCCT1). A comparative analysis showed that retinol dose-dependently inhibits the growth of these four types of resistant cancer cells, while retinoic acid and retinyl palmitate have a limited effect. Also, the adhesion of these cells in vitro was inhibited by retinol treatment, more effectively than by retinoic acid (except for the JHP-1 cell line) [126,127]. In a review article by Zhang et al. [128], the presented data support the role of retinoic acid as a growth suppressor of ovarian cancer cells. Retinoid-binding (RB) family proteins, particularly RB2/p130, are molecular targets responsible for the retinoid-mediated growth inhibition of ovarian cancer cells. Yilmaz et al. [129] described the evolution of acute promyelocytic leukemia (APL) therapy, including the use of all-*trans* retinoic acid as an effective contributor to symptom reduction. All-*trans* retinoic acid, involving RXRs and RARs, is effective in the treatment of acute promyelocytic leukemia [117]. All-*trans* retinoic acid’s action against acute promyelocytic leukemia is probably related to promoting the transcriptional activation of genes related to the differentiation and regulation of autophagy through the inhibition of the mammalian target of rapamycin (mTOR) [130]. All-*trans* retinoic acid inhibits human pancreatic ductal adenocarcinoma (MiaPaCa-2) proliferation and migration, which is associated with the downregulation of p21-activated kinase (PAK) [131]. Also, PAK1 deprivation increases all-*trans* retinoic acid sensitivity [131]. Terao et al. [132] studied the effects of all-*trans* retinoic acid on breast cancer cells. In this study [132], the anti-cancer activity of all-*trans* retinoic acid was obtained due to a reduction in the mitochondrial respiration/energy balance deficits (reduced expression of nuclear genes encoding mitochondrial proteins) of breast cancer cells. Li et al. [133] have shown that retinol is a potent antiproliferative agent against human treatment-resistant cancers, including gallbladder cancer. The study additionally explored how vitamin A affected the growth of human gallbladder cancer cells in vivo. It was found that serum retinol levels were significantly lower in xenograft mice with tumors derived from various treatment-resistant cancer cell lines compared to control mice [133]. Another research group [134] has shown that 13-*cis* retinoic acid induces tumor cell differentiation by reducing extracellular matrix (ECM) stiffness and increasing neurite hypertrophy. The extracellular matrix significantly influences tumor behavior, including proliferation, invasion, and metastasis. Meanwhile, the differentially altered proteins identified in this study may be new biomarkers of the drug’s efficacy in treating neuroblastoma [134]. Another review examined the relationship between vitamin A metabolism and the extracellular matrix in tumors [135]. Vitamin A derivatives show potential in modulating extracellular matrix changes (transcriptional regulation, extracellular matrix components, and matrix metalloproteinases). Vitamin A and its derivatives may be used as supplementary medications in cancer treatment in the future since they influence tumor dynamics [135]. However, the clinical usage of retinol is still limited due to the difficulties in converting these benefits into therapeutic results [135].

However, clinical and preclinical study results are less promising when it comes to vitamin A or its derivatives preventing or treating cancer. In a phase II study (clinical data) of 13-*cis*retinoic acid in combination with gemcitabine in patients with inoperable pancreatic cancer (aged 44 to 70), no improvement was observed [136]. Further clinical studies [137,138] have demonstrated the effect of 13-*cis* retinoic acid in patients with neuroblastoma (phase III trial). For this purpose, long-term treatment effects (5 years) and the benefits of 13-*cis*retinoic acid supplementation were monitored. Although the overall difference in patient survival after 5 years was not statistically significant [137,138], an increase in the 5-year survival rate was observed in the group of patients with minimal residual disease [138]. In subsequent studies [139] of patients with advanced stages of neuroblastoma (stages 3 and 4), no benefit was obtained from additional supplementation with 13-*cis*retinoic acid. Adamson et al. [140] reported that all-*trans* retinoic acid and IFN-α2a was inactive in children with relapsed or refractory neuroblastoma and Wilms tumors.

Subsequent clinical trials [141] examined the effects of various antioxidants, including vitamin A, on breast cancer patients before and after surgery (data obtained from two hospitals in Sweden). Using vitamin A and other antioxidants after surgery yields a favorable prognosis. In the preoperative setting, doubling the aryl hydrocarbon receptor (AhR) was achieved, which may influence tumor activation. Table 1 illustrates a systematic summary of the effects of vitamin A and its derivatives in the context of pro- or anti-cancer activity. The summary includes information on the population, doses used, study design, and conclusions drawn from the conducted research.

It should be noted that in studies [136,140], the research was conducted on a small number of people of both male and female genders. In contrast, in studies [137,138,139,141], the population is large, but covers a wide age range (e.g., children aged 1–18), both sexes, and different stages of cancer. The cited clinical studies cannot be directly compared, but they constitute a preliminary form of monitoring the impact of vitamin A derivatives on tumorigenesis. The data compiled in Table 1 may contribute to determining a potential path for future clinical trials, including reducing the heterogeneity of the study design. In parallel, it is worth emphasizing that no toxic effects of vitamin A derivatives were found in any of the cited studies.

Also, the literature includes studies considering the use of carotenoids, e.g., β-carotene in cancer prevention/treatment. In 2010 [142], the effect of β-carotene supplementation was analyzed in terms of the incidence of colorectal cancer, pancreatic cancer, prostate cancer, breast cancer, melanoma, and non-melanoma skin cancer; however, no significant result was observed. Supplementing with β-carotene at a daily dosage of 20–30 mg significantly enhanced the risk of lung and stomach malignancies [142]. In the following years [143], some studies showed that higher plasma concentrations of β-carotene and α-carotene were associated with a lower risk of breast cancer in estrogen receptor-negative tumors. Also, carotenoid intake in pancreatic cancer was analyzed [144]. The opposite relationship was discovered between β-carotene consumption and pancreatic cancer incidence [144]. An association was identified between a high risk of liver cancer (hepatitis C virus (HCV), early stage) and low serum β-carotene levels [145].

## 7. Daily Requirement of Vitamin A

A daily intake of vitamin A needs to be provided for the body to function properly. According to the 2015 data of the European Food Safety Authority (EFSA) [146], the Population Reference Intakes (PRIs) are 750 µg retinol equivalent/day for men and 650 µg retinol equivalent/day for women. By definition, 1 μg of retinol equivalent is equivalent to either 1 μg of retinol, 6 μg of β-carotene, or 12 μg of other provitamin A carotenoids. The retinol-equivalent values are much higher for pregnancy and lactation, at 700 and 1300 µg retinol equivalent/day, respectively. This is because it takes into account the additional vitamin A requirements associated with retinol accumulation in fetal and maternal tissues, as well as retinol transfer to breast milk. The Population Reference Intakes for infants and children, depending on age and weight, range from 250 to 750 µg retinol equivalent/day. In 2024, the EFSA issued a scientific opinion [147] that determined the tolerable upper intake level (UL) for preformed vitamin A. The upper intake level value for men and women, including women of child-bearing age, pregnant and lactating women, and postmenopausal women, is 3000 μg retinol equivalent/day.

## 8. Conclusions

Although vitamin A was discovered as early as 1913, the full scope of its biological activity has still not been fully explored. In the past few decades, there have been many studies and articles focusing on the effects of carotenoids and vitamin A, and its derivatives both in vitro and in clinical trials.

Many review articles have focused on the action of carotenoids, vitamin A, and its derivatives, as well as their functions, absorption, and transport to target tissues. This article provides a summary of pertinent studies and results, reflecting the current state of research on the antioxidant properties of carotenoids, vitamin A, and their derivatives. This review article compiles the recent findings concerning carotenoids’ and vitamin A’s impact on DNA repair. The obtained conclusions are outlined in the following points:

- Anti- vs. pro-oxidant activities of carotenoids, vitamin A, and its derivatives are discussed, mostly based on studies that use physiologically relevant reactive species for oxidative stress;
- The active metabolites of vitamin A, especially retinoic acid, affect the expression of the genes responsible for genome stability and DNA repair. It can be hypothesized that vitamin A has different effects depending on the type of retinoic acid receptor that undergoes dominant expression in a specific tissue. The role of vitamin A and its derivatives in DNA repair has been particularly well demonstrated in vitro and in experimental animals. Additionally, certain clinical studies argue that vitamin A, certain carotenoids, or their mixtures reduce DNA damage;
- The non-nuclear action of retinoic acid, in which RARs interact at the cell membrane, has been indicated. RARs present in lipid rafts, interact with Gαq protein, and thus activate the kinase pathway;
- Carotenoids, vitamin A, and its derivatives exhibit anti-cancer properties; however, the cited literature mainly refers to in vitro studies. In the clinical trials conducted so far, there has been no clear answer as to whether carotenoids, vitamin A, or its derivatives could reduce the risk of cancer or be used in treatment against cancer;
- In the future, the use of retinoids may be an effective therapy in the fight against cancer. Yet, further research is needed to develop it to ensure the required efficacy of these substances while maintaining safety for the human body;
- Vitamin A should be supplemented as recommended by the European Food Safety Authority. On the other hand, the consumption of vitamin A above the tolerable upper intake level can result in teratogenicity and hepatotoxicity [147].

Vitamin A is one of the most important vitamins due to its functions. The current pace of life, especially in highly developed countries, increases the consumption of heavily processed foods. This causes a reduction in vitamins in the diet, especially vitamin A, and its deficiency can lead to severe health issues. Vitamin A insufficiency is also a concern in underdeveloped nations [148], due to restricted availability, low material status, and a lack of information about the importance of vitamin and mineral supplementation in the diet. Implementing suitable dietary strategies and programs that primarily target children, pregnant and lactating women, and the elderly will minimize the risk of a variety of diseases, lessening the strain on healthcare systems.

## Figures and Tables

**Table 1:** The systematic synthesis of vitamin A and its derivatives’ role studied in the clinical trials.

Population	Intervention and Study Design	Outcomes	Source
Metastatic pancreatic cancer patients—15 men and 15 women aged from 44 to 79 years.	Gemcitabine 1000 mg/m^2^ on days 8, 15, and 22 plus 13-*cis* retinoic acid 1 mg/kg on days 1–14 for six cycles.	Well tolerated; no improvement in the response rate.	Report of the Phase II Trials [136].
Children with neuroblastoma aged from 1 to 18 years: 434 patients had Evans stage IV neuroblastoma; 72 had stage III; 1 had stage II; 13 had stage I or II with bone metastases; 19 had stage IV MYCN amplification, with an age of less than 1 year.	Two randomizations: transplantation vs. continuous chemotherapy and 13-*cis* retinoic acid vs. no further treatment.	The 3-year event-free survival rate was significantly higher in patients receiving 13-*cis* retinoic acid; overall survival was not significantly different.	Multi-center study of the Children’s Cancer Group [137].
539 child patients aged from 1 to 18 years that were randomly assigned to consolidation with myeloablative chemotherapy, total-body irradiation and autologous purged bone marrow transplantation (379 patients), or three cycles of intensive chemotherapy (258 patients), and subsequent treatment with 13-*cis* retinoic acid.	Patients who completed consolidation without disease progression were randomly assigned to receive no further therapy or 13-*cis* retinoic acid for 6 months—six cycles of 160 mg/m^2^/day in two divided doses for 14 days every 28 days.	The 5-year event-free survival was higher for 13-*cis*retinoic acid (not statistically significant). The 5-year overall survival was higher for patients supplemented with 13-*cis* retinoic acid.	Children’s oncology group study [138].
175 neuroblastoma patients—children (stage III or IV).	Supplementation of 13-*cis* retinoic acid at a dose of 0.75 mg/kg/day or placebo for up to 4 years.	Mild toxicity, no advantage in event-free survival.	[139]
17 patients with neuroblastoma (median age 9 years) and 15 patients with Wilms tumor (median age 6 years).	A phase II trial of all-*trans* retinoic acid at a dose of 90 mg/m^2^/day in three divided doses for 3 consecutive days per week, and IFN-α2a of 3 × 10^6^ U/m^2^/day for 5 consecutive days per week, in 4-week cycles.	The combination of all-*trans*-retinoic acid and IFN-α2a was inactive in children with relapsed or refractory neuroblastoma and Wilms tumor.	A Pediatric Oncology Branch, NCI and Children’s Oncology Group Study [140].
2333 breast cancer patients.	Supplementation of vitamin A before and during chemotherapy; pre- and postoperative.	Before and during chemotherapy—worse prognosis.Pre- and postoperative prognosis significantly different depending on AhRnuc status.	Clinical data obtained from patient charts [141].

## Abbreviations

The following abbreviations are used in this manuscript:

AP	Acute pancreatitis
ARAT	Acyl-CoA acyl transferase
ATRA	All-*trans* retinoic acid
CAR	Carotenoids
CAT	Catalase
CM	Chylomicron
CMR	Chylomicron remnants
CM-RE	Chylomicron remnants–retinyl ester
CRABP	Cellular retinoic acid-binding protein
CRBP	Cellular retinol-binding protein
CP	Chronic pancreatitis
ECM	Extracellular matrix
EFSA	European Food Safety Authority
GSH-px	Glutathione peroxidase
LRAT	Lecithin retinol acyl transferase
MAPK	Mitogen-activated protein kinase
MMP	Matrix metalloproteinase
MDA	Malondialdehyde
RA	Retinoic acid
RALDH	Retinaldehyde dehydrogenase
RE	Retinyl ester
RDH	Retinol dehydrogenases
ROH	Retinol
RAR	Retinoic acid receptor
RBP	Retinol-binding protein
RXR	Retinoid X receptor
ROS	Reactive oxygen species
SOD	Superoxide dismutase
SSBs/DSBs	Single/double-strand breaks

## References

[1] Carazo A., Macáková K., Matoušová K., Krčmová L.K., Protti M., Mladěnka P. (2021). Vitamin a Update: Forms, Sources, Kinetics, Detection, Function, Deficiency, Therapeutic Use and Toxicity. Nutrients.

[2] Molavi F., Sarabi-Aghdam V., Mirarab Razi S., Rashidinejad A. (2022). Vitamin A. Handbook of Food Bioactive Ingredients.

[3] Edem D.O. (2009). Vitamin A: A Review. Asian J. Clin. Nutr..

[4] Menezes M.S.S., Almeida C.M.M. (2024). Structural, Functional, Nutritional and Clinical Aspects of Vitamin A: A Review. PharmaNutrition.

[5] D’Ambrosio D.N., Clugston R.D., Blaner W.S. (2011). Vitamin A Metabolism: An Update. Nutrients.

[6] Maiani G., Castón M.J.P., Catasta G., Toti E., Cambrodón I.G., Bysted A., Granado-Lorencio F., Olmedilla-Alonso B., Knuthsen P., Valoti M. (2009). Carotenoids: Actual Knowledge on Food Sources, Intakes, Stability and Bioavailability and Their Protective Role in Humans. Mol. Nutr. Food Res..

[7] Kelly M.E., Ramkumar S., Sun W., Colon Ortiz C., Kiser P.D., Golczak M., Von Lintig J. (2018). The Biochemical Basis of Vitamin A Production from the Asymmetric Carotenoid β-Cryptoxanthin. ACS Chem. Biol..

[8] Prathyusha P., Viswanathan G., Tomcy A.T., Binitha P.P., Bava S.V., Sindhu E.R. (2025). Lutein and inflammation: A comprehensive review of its mechanisms of action. Explor. Drug Sci..

[9] Kapała A., Szlendak M., Motacka E. (2022). The Anti-Cancer Activity of Lycopene: A Systematic Review of Human and Animal Studies. Nutrients.

[10] Sajovic J., Meglič A., Glavač D., Markelj Š., Hawlina M., Fakin A. (2022). The Role of Vitamin A in Retinal Diseases. Int. J. Mol. Sci..

[11] Huang Z., Liu Y., Qi G., Brand D., Zheng S.G. (2018). Role of Vitamin A in the Immune System. J. Clin. Med..

[12] Valdes A.M., Louca P., Visconti A., Asnicar F., Bermingham K., Nogal A., Wong K., Michelotti G.A., Wolf J., Segata N. (2024). Vitamin A Carotenoids, but Not Retinoids, Mediate the Impact of a Healthy Diet on Gut Microbial Diversity. BMC Med..

[13] Kiser P.D., Palczewski K. (2021). Pathways and Disease-Causing Alterations in Visual Chromophore Production for Vertebrate Vision. J. Biol. Chem..

[14] von Lintig J., Kiser P.D., Golczak M., Palczewski K. (2010). The Biochemical and Structural Basis for Trans-to-Cis Isomerization of Retinoids in the Chemistry of Vision. Trends Biochem. Sci..

[15] Palczewski K. (2006). G Protein–Coupled Receptor Rhodopsin. Annu. Rev. Biochem..

[16] Zhong M., Kawaguchi R., Kassai M., Sun H. (2012). Retina, Retinol, Retinal and the Natural History of Vitamin A as a Light Sensor. Nutrients.

[17] Norsa L., Zazzeron L., Cuomo M., Claut L., Bulfamante A.M.C., Biffi A., Colombo C. (2019). Night Blindness in Cystic Fibrosis: The Key Role of Vitamin A in the Digestive System. Nutrients.

[18] Shim E., Yeum K.J., Tang G., Ahn S.H., Hwang J., Lee-Kim Y.C. (2012). Retinoids, Carotenoids, and Tocopherols in Breast Adipose Tissue and Serum of Benign Breast Disease and Breast Cancer Patients. Nutr. Cancer.

[19] Hadi H., Stoltzfus R.J., Dibley M.J., Moulton L.H., West K.P., Kjolhede C.L., Sadjimin T. (2000). Vitamin A Supplementation Selectively Improves the Linear Growth of Indonesian Preschool Children: Results from a Randomized Controlled Trial. Am. J. Clin. Nutr..

[20] Ross A.C., Gardner E.M. (1994). The Function of Vitamin A in Cellular Growth and Differentiation, and Its Roles during Pregnancy and Lactation. Adv. Exp. Med. Biol..

[21] Bradley E.J., Griffiths C.E.M., Sherratt M.J., Bell M., Watson R.E.B. (2015). Over-the-Counter Anti-Ageing Topical Agents and Their Ability to Protect and Repair Photoaged Skin. Maturitas.

[22] Schagen S.K., Zampeli V.A., Makrantonaki E., Zouboulis C.C. (2012). Discovering the Link between Nutrition and Skin Aging. Dermatoendocrinol.

[23] Oblong J.E., Jansen J.H. (2022). Topical Vitamins. Cosmetic Dermatology: Products and Procedures.

[24] Michalak M., Pierzak M., Kręcisz B., Suliga E. (2021). Bioactive Compounds for Skin Health: A Review. Nutrients.

[25] Quan T. (2023). Human Skin Aging and the Anti-Aging Properties of Retinol. Biomolecules.

[26] Reboul E. (2013). Absorption of Vitamin A and Carotenoids by the Enterocyte: Focus on Transport Proteins. Nutrients.

[27] Chen W., Chen G. (2014). The Roles of Vitamin A in the Regulation of Carbohydrate, Lipid, and Protein Metabolism. J. Clin. Med..

[28] Blomhoff R. (1994). Transport and Metabolism of Vitamin A. Nutr. Rev..

[29] Dela Seña C., Riedl K.M., Narayanasamy S., Curley R.W., Schwartz S.J., Harrison E.H. (2014). The Human Enzyme That Converts Dietary Provitamin A Carotenoids to Vitamin A Is a Dioxygenase. J. Biol. Chem..

[30] Harrison E.H. (2012). Mechanisms Involved in the Intestinal Absorption of Dietary Vitamin A and Provitamin A Carotenoids. Biochim. Biophys. Acta Mol. Cell Biol. Lipids.

[31] Blaner W.S., Li Y., Brun P.J., Yuen J.J., Lee S.A., Clugston R.D. (2016). Vitamin A Absorption, Storage and Mobilization. Subcell. Biochem..

[32] Senoo H., Kojima N., Sato M. (2007). Vitamin A-Storing Cells (Stellate Cells). Vitam. Horm..

[33] Kedishvili N.Y. (2013). Enzymology of Retinoic Acid Biosynthesis and Degradation. J. Lipid Res..

[34] Belyaeva O.V., Adams M.K., Popov K.M., Kedishvili N.Y. (2020). Generation of Retinaldehyde for Retinoic Acid Biosynthesis. Biomolecules.

[35] Palace V.P., Khaper N., Qin Q., Singal P.K. (1999). Antioxidant Potentials of Vitamin A and Carotenoids and Their Relevance to Heart Disease. Free Radic. Biol. Med..

[36] Noy N., Blaner W.S. (1991). Interactions of Retinol with Binding Proteins: Studies with Rat Cellular Retinol-Binding Protein and with Rat Retinol-Binding Protein. Biochemistry.

[37] Zizola C.F., Frey S.K., Jitngarmkusol S., Kadereit B., Yan N., Vogel S. (2010). Cellular Retinol-Binding Protein Type I (CRBP-I) Regulates Adipogenesis. Mol. Cell Biol..

[38] Ciaccio M., Valenza M., Tesoriere L., Bongiorno A., Albiero R., Livrea M.A. (1993). Vitamin A Inhibits Doxorubicin-Induced Membrane Lipid Peroxidation in Rat Tissues In Vivo. Arch. Biochem. Biophys..

[39] De Boeck H., Zidovetzki R. (1988). NMR Study of the Interaction of Retinoids with Phospholipid Bilayers. Biochim. Biophys. Acta (BBA)-Biomembr..

[40] During A., Harrison E.H. (2007). Mechanisms of Provitamin A (Carotenoid) and Vitamin A (Retinol) Transport into and out of Intestinal Caco-2 Cells. J. Lipid Res..

[41] Jung U.S., Jeong K.J., Kang J.K., Yi K., Shin J.H., Seo H.S., Kim T., Kim S.H., Hur J.Y. (2013). Effects of Estrogen Receptor α and β on the Expression of Visfatin and Retinol-Binding Protein 4 in 3T3-L1 Adipocytes. Int. J. Mol. Med..

[42] Clugston R.D., Blaner W.S. (2012). The Adverse Effects of Alcohol on Vitamin A Metabolism. Nutrients.

[43] Sies H., Jones D.P. (2020). Reactive Oxygen Species (ROS) as Pleiotropic Physiological Signalling Agents. Nat. Rev. Mol. Cell Biol..

[44] De Almeida A.J.P.O., De Oliveira J.C.P.L., Da Silva Pontes L.V., De Souza Júnior J.F., Gonçalves T.A.F., Dantas S.H., De Almeida Feitosa M.S., Silva A.O., De Medeiros I.A. (2022). ROS: Basic Concepts, Sources, Cellular Signaling, and Its Implications in Aging Pathways. Oxid. Med. Cell Longev..

[45] Bernatoniene J., Kopustinskiene D.M. (2018). The Role of Catechins in Cellular Responses to Oxidative Stress. Molecules.

[46] Palma F.R., Gantner B.N., Sakiyama M.J., Kayzuka C., Shukla S., Lacchini R., Cunniff B., Bonini M.G. (2024). ROS Production by Mitochondria: Function or Dysfunction?. Oncogene.

[47] Tirichen H., Yaigoub H., Xu W., Wu C., Li R., Li Y. (2021). Mitochondrial Reactive Oxygen Species and Their Contribution in Chronic Kidney Disease Progression Through Oxidative Stress. Front. Physiol..

[48] Pizzino G., Irrera N., Cucinotta M., Pallio G., Mannino F., Arcoraci V., Squadrito F., Altavilla D., Bitto A. (2017). Oxidative Stress: Harms and Benefits for Human Health. Oxid. Med. Cell Longev..

[49] Galkina O.V. (2013). The Specific Features of Free-Radical Processes and the Antioxidant Defense in the Adult Brain. Neurochem. J..

[50] Didier A.J., Stiene J., Fang L., Watkins D., Dworkin L.D., Creeden J.F. (2023). Antioxidant and Anti-Tumor Effects of Dietary Vitamins A, C, and E. Antioxidants.

[51] Lobo V., Patil A., Phatak A., Chandra N. (2010). Free Radicals, Antioxidants and Functional Foods: Impact on Human Health. Pharmacogn. Rev..

[52] El-Agamey A., Lowe G.M., McGarvey D.J., Mortensen A., Phillip D.M., Truscott T.G., Young A.J. (2004). Carotenoid Radical Chemistry and Antioxidant/pro-Oxidant Properties. Arch. Biochem. Biophys..

[53] Tesoriere L., Ciaccio M., Bongiorno A., Riccio A., Pintaudi A.M., Livrea M.A. (1993). Antioxidant Activity of All-Trans-Retinol in Homogeneous Solution and in Phosphatidylcholine Liposomes. Arch. Biochem. Biophys..

[54] Dao D.Q., Ngo T.C., Thong N.M., Nam P.C. (2017). Is Vitamin A an Antioxidant or a Pro-Oxidant?. J. Phys. Chem. B.

[55] Kayalar O., Bayrak B.B., Yildirim M., Yanardag R., Oztay F. (2024). Retinoic Acid Reduces Kidney Injury by Regulating Oxidative Stress, NRF-2, and Apoptosis in Hyperoxic Mice. Cell Biochem. Funct..

[56] Iqbal A., Kamran S.H., Siddique F., Ishtiaq S., Hameed M., Manzoor M. (2024). Modulatory Effects of Rutin and Vitamin A on Hyperglycemia Induced Glycation, Oxidative Stress and Inflammation in High-Fat-Fructose Diet Animal Model. PLoS ONE.

[57] Wu P., Shen N., Feng S., Liu W., Wang J., Wang C. (2024). Oxidative Stress and Apoptosis of the Spinal Cord in a Rat Model of Retinoic Acid-Induced Neural Tube Defects. Int. J. Dev. Neurosci..

[58] Milani A., Basirnejad M., Shahbazi S., Bolhassani A. (2017). Carotenoids: Biochemistry, Pharmacology and Treatment. Br. J. Pharmacol..

[59] Farhana A., Khan Y.S., Alsrhani A., Manni E., Alameen A.A.M., Derafa W., Alhathlaul N., Atif M., Eltayeb L.B. (2025). Antioxidant and Prooxidant Functions of Carotenoids in Human Health: Trigger Factors, Mechanism and Application. Recent Advances in Phytochemical Research.

[60] Young A.J., Lowe G.M. (2001). Antioxidant and Prooxidant Properties of Carotenoids. Arch. Biochem. Biophys..

[61] Fiedor J., Burda K. (2014). Potential Role of Carotenoids as Antioxidants in Human Health and Disease. Nutrients.

[62] Siems W., Wiswedel I., Salerno C., Crifo C., Augustin W., Schild L., Langhans C.D., Sommerburg O. (2005). Beta-carotene breakdown products may impair mitochondrial functions—Potential side effects of high-dose beta-carotene supplementation. J. Nutr. Biochem..

[63] Charlton N.C., Mastyugin M., Török B., Török M. (2023). Structural Features of Small Molecule Antioxidants and Strategic Modifications to Improve Potential Bioactivity. Molecules.

[64] Pérez-Gálvez A., Viera I., Roca M. (2020). Carotenoids and Chlorophylls as Antioxidants. Antioxidants.

[65] Salerno C., Crifo C., Capuozzo E., Sommerburg O., Langhans C.D., Siems W. (2005). Effect of carotenoid oxidation products on neutrophil viability and function. Biofactors.

[66] Siems W., Capuozzo E., Crifo C., Sommerburg O., Langhans C.D., Schlipalius L., Wiswedel I., Kraemer K., Salerno C. (2003). Carotenoid cleavage products modify respiratory burst and induce apoptosis of human neutrophils. BBA-Mol. Basis Dis..

[67] Kikugawa K., Hiramoto K., Tomiyama S., Asano Y. (1997). β-Carotene effectively scavenges toxic nitrogen oxides: Nitrogen dioxide and peroxynitrous acid. FEBS Lett..

[68] Sen Gupta S., Ghosh M. (2013). In vitro antioxidative evaluation of α- and β-carotene, isolated from crude palm oil. J. Anal. Methods Chem..

[69] Friedberg E.C., McDaniel L.D., Schultz R.A. (2004). The Role of Endogenous and Exogenous DNA Damage and Mutagenesis. Curr. Opin. Genet. Dev..

[70] Swenberg J.A., Lu K., Moeller B.C., Gao L., Upton P.B., Nakamura J., Starr T.B. (2010). Endogenous versus Exogenous DNA Adducts: Their Role in Carcinogenesis, Epidemiology, and Risk Assessment. Toxicol. Sci..

[71] Evans M.D., Dizdaroglu M., Cooke M.S. (2004). Oxidative DNA Damage and Disease: Induction, Repair and Significance. Mutat. Res. Rev. Mutat. Res..

[72] Cooke M.S., Evans M.D., Dizdaroglu M., Lunec J. (2003). Oxidative DNA Damage: Mechanisms, Mutation, and Disease. FASEB J..

[73] Chandimali N., Bak S.G., Park E.H., Lim H.J., Won Y.S., Kim E.K., Park S.I., Lee S.J. (2025). Free Radicals and Their Impact on Health and Antioxidant Defenses: A Review. Cell Death Discov..

[74] Inoue M., Sato E.F., Nishikawa M., Park A.-M., Kira Y., Imada I., Utsumi K. (2005). Mitochondrial Generation of Reactive Oxygen Species and Its Role in Aerobic Life. Curr. Med. Chem..

[75] Cadet J., Wagner J.R. (2013). DNA Base Damage by Reactive Oxygen Species, Oxidizing Agents, and UV Radiation. Cold Spring Harb. Perspect. Biol..

[76] Kehrer J.P. (2000). The Haber-Weiss Reaction and Mechanisms of Toxicity. Toxicology.

[77] Al Tanoury Z., Piskunov A., Rochette-Egly C. (2013). Vitamin a and Retinoid Signaling: Genomic and Nongenomic Effects. J. Lipid Res..

[78] Takahashi N., Saito D., Hasegawa S., Yamasaki M., Imai M. (2022). Vitamin A in Health Care: Suppression of Growth and Induction of Differentiation in Cancer Cells by Vitamin A and Its Derivatives and Their Mechanisms of Action. Pharmacol. Ther..

[79] Piskunov A., AI Tanoury Z., Rochette-Egly C. (2014). Nuclear and Extra-Nuclear Effects of Retinoid Acid Receptors: How They Are Interconnected. Subcell. Biochem..

[80] Le Maire A., Teyssier C., Balaguer P., Bourguet W., Germain P. (2019). Regulation of RXR-RAR Heterodimers by RXR- and RAR-Specific Ligands and Their Combinations. Cells.

[81] Talib W.H., Ahmed Jum’AH D.A., Attallah Z.S., Jallad M.S., Al Kury L.T., Hadi R.W., Mahmod A.I. (2023). Role of Vitamins A, C, D, E in Cancer Prevention and Therapy: Therapeutic Potentials and Mechanisms of Action. Front. Nutr..

[82] Loinder K., Söderström M. (2003). The Nuclear Receptor Corepressor (N-CoR) Modulates Basal and Activated Transcription of Genes Controlled by Retinoic Acid. J. Steroid Biochem. Mol. Biol..

[83] Vašková J., Stupák M., Vidová Ugurbaş M., Židzik J., Mičková H. (2025). Therapeutic Uses of Retinol and Retinoid-Related Antioxidants. Molecules.

[84] Burzyński J., Fichna J., Tarasiuk A. (2023). Putative Molecular Targets for Vitamin A in Neutralizing Oxidative Stress in Acute and Chronic Pancreatitis—A Systematic Review. Naunyn Schmiedebergs Arch. Pharmacol..

[85] Baldwin H.E., Nighland M., Kendall C., Mays D.A., Grossman R., Newburger J. (2013). 40 Years of Topical Tretinoin Use in Review. J. Drugs Dermatol..

[86] Xu A., Zhang N., Cao J., Zhu H., Yang B., He Q., Shao X., Ying M. (2020). Post-Translational Modification of Retinoic Acid Receptor Alpha and Its Roles in Tumor Cell Differentiation. Biochem. Pharmacol..

[87] Catalanotto C., Cogoni C., Zardo G. (2016). MicroRNA in Control of Gene Expression: An Overview of Nuclear Functions. Int. J. Mol. Sci..

[88] Gilardi F., Desvergne B. (2014). The Biochemistry of Retinoic Acid Receptors I: Structure, Activation, and Function at the Molecular Level. Subcell. Biochem..

[89] Vergoulis T., Vlachos I.S., Alexiou P., Georgakilas G., Maragkakis M., Reczko M., Gerangelos S., Koziris N., Dalamagas T., Hatzigeorgiou A.G. (2011). TarBase 6.0: Capturing the exponential growth of miRNA targets with experimental support. Nucleic Acids Res..

[90] Kim D., Kim Y., Kim Y. (2019). Effects of β-carotene on Expression of Selected MicroRNAs, Histone Acetylation, and DNA Methylation in Colon Cancer Stem Cells. Cancer Prev..

[91] Abrego-Guandique D.M., Galmés S., García-Rodríguez A., Caroleo M.C., Ribot J., Cione E., Bonet M.L. (2024). β-Carotene Impacts the Liver MicroRNA Profile in a Sex-Specific Manner in Mouse Offspring of Western Diet-Fed Mothers: Results from Microarray Analysis by Direct Hybridization. Int. J. Mol. Sci..

[92] Evans R.M., Mangelsdorf D.J. (2014). Nuclear Receptors, RXR, and the Big Bang. Cell.

[93] Li Y., Wongsiriroj N., Blaner W.S. (2014). The Multifaceted Nature of Retinoid Transport and Metabolism. Hepatobiliary Surg. Nutr..

[94] Napoli J.L. (2012). Physiological Insights into All-Trans-Retinoic Acid Biosynthesis. Biochim. Biophys. Acta Mol. Cell Biol. Lipids.

[95] Van Loo-Bouwman C.A., Naber T.H.J., Schaafsma G.A. (2014). Review of Vitamin A Equivalency of β-Carotene in Various Food Matrices for Human Consumption. Br. J. Nutr..

[96] Cheung Y.T., Lau W.K.W., Yu M.S., Lai C.S.W., Yeung S.C., So K.F., Chang R.C.C. (2009). Effects of All-Trans-Retinoic Acid on Human SH-SY5Y Neuroblastoma as in Vitro Model in Neurotoxicity Research. Neurotoxicology.

[97] Masiá S., Alvarez S., De Lera A.R., Barettino D. (2007). Rapid, Nongenomic Actions of Retinoic Acid on Phosphatidylinositol-3-Kinase Signaling Pathway Mediated by the Retinoic Acid Receptor. Mol. Endocrinol..

[98] Pan J., Kao Y.L., Joshi S., Jeetendran S., DiPette D., Singh U.S. (2005). Activation of Rac1 by Phosphatidylinositol 3-Kinase In Vivo: Role in Activation of Mitogen-Activated Protein Kinase (MAPK) Pathways and Retinoic Acid-Induced Neuronal Differentiation of SH-SY5Y Cells. J. Neurochem..

[99] Poetsch A.R. (2020). The Genomics of Oxidative DNA Damage, Repair, and Resulting Mutagenesis. Comput. Struct. Biotechnol. J..

[100] Moris D., Spartalis M., Spartalis E., Karachaliou G.S., Karaolanis G.I., Tsourouflis G., Tsilimigras D.I., Tzatzaki E., Theocharis S. (2017). The Role of Reactive Oxygen Species in the Pathophysiology of Cardiovascular Diseases and the Clinical Significance of Myocardial Redox. Ann. Transl. Med..

[101] Fei J., Demillard L.J., Ren J. (2022). Reactive Oxygen Species in Cardiovascular Diseases: An Update. Explor. Med..

[102] Nakamura H., Takada K. (2021). Reactive Oxygen Species in Cancer: Current Findings and Future Directions. Cancer Sci..

[103] Astley S.B., Elliott R.M., Archer D.B., Southon S. (2004). Evidence That Dietary Supplementation with Carotenoids and Carotenoid-Rich Foods Modulates the DNA Damage: Repair Balance in Human Lymphocytes. Br. J. Nutr..

[104] Park E., Park Y.K., Kim S.M., Lee H.J., Kang M.H. (2009). Susceptibility to oxidative stress is greater in Korean patients with coronary artery disease than healthy subjects. J. Clin. Biochem. Nutr..

[105] Caliskan-Can E., Firat H., Ardic S., Simsek B., Torun M., Yardim-Akaydin S. (2008). Increased levels of 8-hydroxydeoxyguanosine and its relationship with lipid peroxidation and antioxidant vitamins in lung cancer. Clin. Chem. Lab. Med..

[106] Lagadu S., Lechevrel M., Sichel F., Breton J., Pottier D., Couderc R., Moussa F., Prevost V.J. (2010). 8-oxo-7,8-dihydro-2′-deoxyguanosine as a biomarker of oxidative damage in oesophageal cancer patients: Lack of association with antioxidant vitamins and polymorphism of hOGG1 and GST. Exp. Clin. Cancer Res..

[107] Diaz-Garcia H., Jenny Vilchis-Gil J., Garcia-Roca P., Klünder-Klünder M., Gomez-Lopez J., Granados-Riveron J.T., Sanchez-Urbina J. (2022). Dietary and Antioxidant Vitamins Limit the DNA Damage Mediated by Oxidative Stress in the Mother-Newborn Binomial. Life.

[108] Loft S., Vistisen K., Ewertz M., Tjønneland A., Overvad K., Poulsen H.E. (1992). Oxidative DNA damage estimated by 8-hydroxydeoxyguanosine excretion in humans: Influence of smoking, gender and body mass index. Carcinogenesis.

[109] Prasad M.P.R., Mukundan M.A., Krishnaswamy K. (1995). Micronuclei and Carcinogen DNA Adducts as Intermediate End Points in Nutrient Intervention Trial of Precancerous Lesions in the Oral Cavity. Eur. J. Cancer B Oral Oncol..

[110] Collins A.R., Olmedilla B., Southon S., Granado F., Duthie S.J. (1998). Serum carotenoids and oxidative DNA damage in human lymphocytes. Carcinogenesis.

[111] Haegele A.D., Gillette C., O’Neill C., Wolfe P., Heimendinger J., Sedlacek S., Thompson H.T. (2000). Plasma xanthophyll carotenoids correlate inversely with indices of oxidative DNA damage and lipid peroxidation. Cancer Epidemiol. Biomark. Prev..

[112] Zhao X., Aldini G., Johnson E.J., Rasmussen H., Kraemer K., Woolf H., Musaeus N., Krinsky N.I., Russell R.M., Yeum K.J. (2006). Modification of lymphocyte DNA damage by carotenoid supplementation in postmenopausal women. Am. J. Clin. Nutr..

[113] Pool-Zobel B.L., Bub A., Muller H., Wollowski I. (1997). Consumption of vegetables reduces genetic damage in humans: First results of a human intervention trial with carotenoid-rich foods. Carcinogenesis.

[114] Velanganni A.A., Dharaneedharan S., Geraldine p., Balasundram C. (2007). Dietary supplementation of vitamin A, C and E prevents p-dimethylaminoazobenzene induced hepatic DNA damage in rats. Indian J. Biochem. Biophys..

[115] Morin B., Narbonne J.F., Ribera D., Badouard C., Ravanat J.L. (2008). Effect of dietary fat soluble vitamins A and E and proanthocyanidin-rich extract from grape seeds on oxidative DNA damage in rats. Food Chem. Toxicol..

[116] Liu C., Bronson R.T., Russell R.M., Wang X.D. (2011). β-Cryptoxanthin supplementation prevents cigarette smoke-induced lung inflammation, oxidative damage, and squamous metaplasia in ferrets. Cancer Prev. Res..

[117] Wolterbeek A.P.M., Roggeband R., van Moorsel C.J.A., Baan R.A., Koeman J.H., Feron V.J., Rutten A.A.J.J.L. (1995). Vitamin A and β-Carotene Influence the Level of Benzo[a]Pyrene-Induced DNA Adducts and DNA-Repair Activities in Hamster Tracheal Epithelium in Organ Culture. Cancer Lett..

[118] Aissa A.F., Lourdes Pires Bianchi M., Carvalho Ribeiro J., Hernandes L.C., Ferreira de Faria A., Zerlotti Mercadante A., Greggi Antunes L.M. (2012). Comparative study of β-carotene and microencapsulated β-carotene: Evaluation of their genotoxic and antigenotoxic effects. Food Chem. Toxicol..

[119] Bhagavathy S., Sumathi P. (2012). Evaluation of antigenotoxic effects of carotenoids from green algae *Chlorococcum humicola* using human lymphocytes. Asian Pac. J. Trop. Biomed..

[120] Murata M., Kawanishi S. (2000). Oxidative DNA Damage by Vitamin A and Its Derivative via Superoxide Generation. J. Biol. Chem..

[121] Lorenzo Y., Azqueta A., Luna L., Bonilla F., Domı’nguez G., Collins A.R. (2009). The carotenoid β-cryptoxanthin stimulates the repair of DNA oxidation damage in addition to acting as an antioxidant in human cells. Carcinogenesis.

[122] Wang K., Zhu X., Zhang K., Zhou F., Zhu L. (2017). Neuroprotective Effect of Tetramethylpyrazine against All-Trans-Retinal Toxicity in the Differentiated Y-79 Cells via Upregulation of IRBP Expression. Exp. Cell Res..

[123] Chen C., Chen J., Wang Y., Liu Z., Wu Y. (2021). Ferroptosis Drives Photoreceptor Degeneration in Mice with Defects in All-Trans-Retinal Clearance. J. Biol. Chem..

[124] Tomaziu-Todosia Anton E., Anton G.I., Scripcariu I.S., Dumitrașcu I., Scripcariu D.V., Balmus I.M., Ionescu C., Visternicu M., Socolov D.G. (2025). Oxidative Stress, Inflammation, and Antioxidant Strategies in Cervical Cancer—A Narrative Review. Int. J. Mol. Sci..

[125] Alexandru I., Nistor D., Motofelea A.C., Cadar B.A., Crintea A., Tatu C., Pop G.N., Csep A.N. (2024). Vitamins, Coenzyme Q10, and Antioxidant Strategies to Improve Oocyte Quality in Women with Gynecological Cancers: A Comprehensive Review. Antioxidants.

[126] Takahashi N. (2022). Inhibitory Effects of Vitamin A and Its Derivatives on Cancer Cell Growth Not Mediated by Retinoic Acid Receptors. Biol. Pharm. Bull..

[127] Li C., Imai M., Matsuura T., Hasegawa S., Yamasaki M., Takahashi N. (2016). Inhibitory Effects of Retinol Are Greater than Retinoic Acid on the Growth and Adhesion of Human Refractory Cancer Cells. Biol. Pharm. Bull..

[128] Zhang D., Holmes W.F., Wu S., Soprano D.R., Soprano K.J. (2000). Retinoids and Ovarian Cancer. J. Cell Physiol..

[129] Yilmaz M., Kantarjian H., Ravandi F. (2021). Acute promyelocytic leukemia current treatment algorithms. Blood Cancer J..

[130] Liang C., Qiao G., Liu Y., Tian L., Hui N., Li J., Ma Y., Li H., Zhao Q., Cao W. (2021). Overview of All-Trans-Retinoic Acid (ATRA) and Its Analogues: Structures, Activities, and Mechanisms in Acute Promyelocytic Leukaemia. Eur. J. Med. Chem..

[131] Wang K., Baldwin G.S., Nikfarjam M., He H. (2019). Antitumor Effects of All-Trans Retinoic Acid and Its Synergism with Gemcitabine Are Associated with Downregulation of P21-Activated Kinases in Pancreatic Cancer. Am. J. Physiol. Gastrointest. Liver Physiol..

[132] Terao M., Goracci L., Celestini V., Kurosaki M., Bolis M., Di Veroli A., Vallerga A., Fratelli M., Lupi M., Corbelli A. (2019). Role of Mitochondria and Cardiolipins in Growth Inhibition of Breast Cancer Cells by Retinoic Acid. J. Exp. Clin. Cancer Res..

[133] Li C., Imai M., Yamasaki M., Hasegawa S., Takahashi N. (2017). Effects of Pre- and Post-Administration of Vitamin A on the Growth of Refractory Cancers in Xenograft Mice. Biol. Pharm. Bull..

[134] Halakos E.G., Connell A.J., Glazewski L., Wei S., Mason R.W. (2019). Bottom up Proteomics Reveals Novel Differentiation Proteins in Neuroblastoma Cells Treated with 13-Cis Retinoic Acid. J. Proteom..

[135] Xie G., Cao S., Wang G., Zhang X., Zhang Y., Wu H., Shen S., Le J., Li K., Huang Z. (2025). Vitamin A and Its Influence on Tumour Extracellular Matrix. Discov. Oncol..

[136] Michael A., Hilly M., Maraveyasz A., Dalgleish A., Lofts F. (2007). 13-cis-Retinoic Acid in Combination with Gemcitabine in the Treatment of Locally Advanced and Metastatic Pancreatic Cancer d Report of a Pilot Phase II Study. Clin. Oncol..

[137] Kurczynski E.M. (2000). Treatment effectiveness of high-risk neuroblastoma is improved with intensive chemotherapy, radiotherapy, autologous bone marrow transplantation, and 13-cis-retinoic acid. Evid.-Based Oncol..

[138] Matthay K.K., Reynolds C.P., Seeger R.C., Shimada H., Adkins E.S., Haas-Kogan D., Gerbing R.B., London W.B., Villablanca J.G. (2009). Long-term results for children with high-risk neuroblastoma treated on a randomized trial of myeloablative therapy followed by 13-cis-retinoic acid: A children’s oncology group study. J. Clin. Oncol..

[139] Kohler J.A., Imeson J., Ellershaw C., Lie S.O. (2000). A randomized trial of 13-Cis retinoic acid in children with advanced neuroblastoma after high-dose therapy. Br. J. Cancer.

[140] Adamson P.C., Matthay K.K., Brien M.O., Reaman G.H., Sato J.K., Balis F.M. (2007). A Phase 2 Trial of All- Trans -Retinoic Acid in Combination with Interferon-α2A in Children with Recurrent Neuroblastoma or Wilms Tumor: A Pediatric Oncology Branch, NCI and Children’s Oncology Group Study. Pediatr. Blood Cancer.

[141] Nilsson L., Khazaei S., Tryggvadottir H., Björner S., Bressan A., Jirström K., Adrian G., Falck A.-K., Borgquist S., Isaksson K. (2023). Pre- and Postoperative Antioxidant Use, Aryl Hydrocarbon Receptor (AhR) Activation and Clinical Outcome in Different Treatment Groups of Breast Cancer Patients. Clin. Breast Cancer.

[142] Druesne-Pecollo N., Latino-Martel P., Norat T., Barrandon E., Bertrais S., Galan P., Hercberg S. (2010). Beta-Carotene Supplementation and Cancer Risk: A Systematic Review and Meta analysis of Randomized Controlled Trials. Int. J. Cancer.

[143] Bakker M.F., Peeters P.H.M., Klaasen V.M., Bueno-De-Mesquita H.B., Jansen E.H.J.M., Ros M.M., Travier N., Olsen A., Tjønneland A., Overvad K. (2016). Plasma Carotenoids, Vitamin C, Tocopherols, and Retinol and the Risk of Breast Cancer in the European Prospective Investigation into Cancer and Nutrition Cohort. Am. J. Clin. Nutr..

[144] Huang X., Gao Y., Zhi X., Ta N., Jiang H., Zheng J. (2016). Association between Vitamin A, Retinol and Carotenoid Intake and Pancreatic Cancer Risk: Evidence from Epidemiologic Studies. Sci. Rep..

[145] Kataria Y., Deaton R.J., Enk E., Jin M., Petrauskaite M., Dong L., Goldenberg J.R., Cotler S.J., Jensen D.M., van Breemen R.B. (2016). Retinoid and Carotenoid Status in Serum and Liver among Patients at High-Risk for Liver Cancer. BMC Gastroenterol..

[146] EFSA NDA Panel (EFSA Panel on Dietetic Products, Nutrition and Allergies) (2015). Scientific Opinion on Dietary Reference Values for Vitamin A. EFSA J..

[147] Turck D., Bohn T., Castenmiller J., de Henauw S., Hirsch-Ernst K.I., Knutsen H.K., Maciuk A., Mangelsdorf I., McArdle H.J., Pentieva K. (2024). Scientific Opinion on the Tolerable Upper Intake Level for Preformed Vitamin A and β-Carotene. EFSA J..

[148] Fernández-Gaxiola A.C., Neufeld L.M., García-Guerra A. (2024). Considerations for Correction of Micronutrient Deficiencies Through Supplementation in Pregnant Women and Children Under-5 in Latin America. Food Nutr. Bull..

